# Comparison of Two Methods for the Measurement of Medial and Lateral Metapodial Bones in Karagouniko Sheep (*Ovis aries*, L. 1758) and Hellenic Goat (*Capra hircus*, L. 1758)

**DOI:** 10.1155/2014/529686

**Published:** 2014-10-30

**Authors:** Aris Pourlis, Theodoros Chatzis, Panagiotis Katsoulos

**Affiliations:** ^1^Laboratory of Anatomy, Histology & Embryology, Faculty of Veterinary Medicine, University of Thessaly, 224 Trikalon Street, 43100 Karditsa, Greece; ^2^Clinic of Farm Animals, Faculty of Veterinary Medicine, Aristotle University of Thessaloniki, 11 St. Voutyra Street, 54627 Thessaloniki, Greece

## Abstract

The objective of this study was to compare the metapodial lengths of sheep and goats measured with a caliper with those measured using a 2-dimensional digital method. Complementarily, the lengths of medial and lateral metapodials in these species were compared. The limbs of 30 ewes and 30 goats were used. After preparation, the lateral and medial length of the metacarpals and metatarsals were measured twice with a caliper. Afterwards, each bone was scanned and the same lengths were digitally measured twice using commercial software. Data analysis revealed strong linear relationship between the two methods but the absolute relative deviation of the measurements with the caliper was significantly higher than those with the 2-dimensional method (*P* < 0.05). All lengths measured with the caliper were significantly higher compared to those measured with the 2-dimensional method (*P* < 0.05). In goats, the lateral length of both metacarpals and metatarsals was significantly higher than medial length (*P* < 0.05); in sheep the lateral length was significantly higher compared to the medial one only in metatarsal bones (*P* < 0.05). In conclusion, the 2-dimensional method is more accurate for the measurement of the metapodials' length than the caliper and there is asymmetry between the medial and lateral metapodials in these species.

## 1. Introduction

Quantitative data of the animal skeleton are extensively used in many biomedical disciplines in order to obtain information of their biology and pathology. The morphological variability of domestic and wild mammals is expressed to a great extent on the skeletal structures [1]. The knowledge of the anatomical differences of the bones may help to elucidate the function and malfunction of body regions such as the limbs [2]. Osteological examination of the excavated bones is very important in terms of identification and study of animal evolution [3].

The metapodial bones of sheep and goat have been used in many studies relating to various scope from zooarchaeology to animal biology and pathology. Additionally, a debate has arisen regarding the contribution of the metapodials in the lameness of the artiodactyls [2, 4]. The previous scientists suggested that the asymmetry observed between the lateral and medial metacarpals and metatarsals of the bovines could be useful for studies on digit function and on the predisposition of cattle to claw disorders.

The commonest tool to measure the bones in terms of longitudinal dimensions is the caliper. On the other hand, many studies employ digital processing of material such as radiographs [4, 5] or photos [2]. The measurement of the length of metapodial bones with a caliper has some difficulties in sheep and goats that arise from the special anatomy of their proximal articular surfaces. So, it is possibly better to use a two-dimensional method for the evaluation of metapodial lengths. The objective of the present study was to compare the measurements obtained in metapodial bones in sheep and goats with a caliper with those obtained by a two-dimensional digital method. A complementary objective was to compare the lengths of medial and lateral metapodials in sheep and goats.

## 2. Materials and Methods

**Figure 1:**
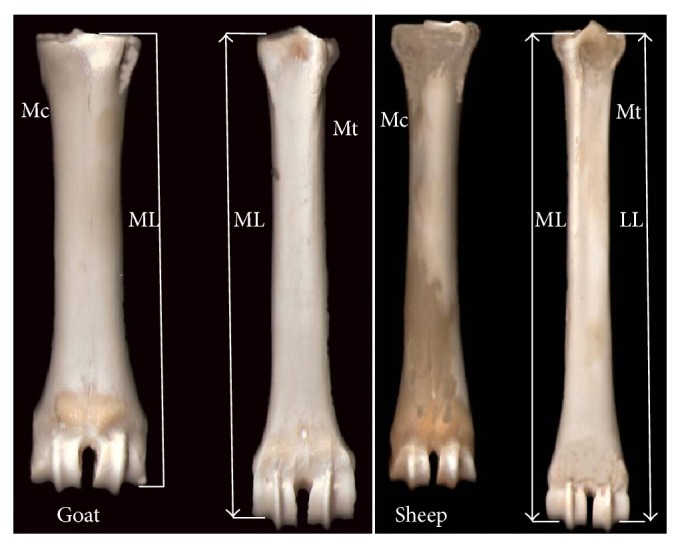
The right metapodial bones of sheep and goat. Mc: metacarpal bone (frontal view); Mt: metatarsal bone (rear view); ML: medial length; LL: lateral length.

The distal extremities of the limbs from 30 ewes and 30 goats (median age 3.7 years) were collected from a slaughterhouse and immediately identified (fore-hind limb) and grouped according to the animal. Consequently, the metapodials were prepared for study by successive boiling, cleaning, degreasing, washing, and drying. At first, the lateral length (LL) and medial length (ML) (Figure 1) of the metacarpals and metatarsals were measured with the aid of caliper (accuracy 0.1 mm) twice by the same researcher (T.C). The variable LL represented the distance from the lateral proximal end of the metapodial bone to the abaxial border of the lateral condyle. Accordingly, the ML represented the distance from the medial proximal end of the metapodial bone to the abaxial border of the medial condyle. Afterwards, each bone was scanned on a scanner (HP scanjet 3670) in order to obtain a digital image of the bone; the same lengths were digitally measured twice by the same person using GIMP 2.0 (GNU Image Manipulation Program) software. Based on these data, the average length for each bone and the absolute relative deviation between the two measurements [|(measurement1 − measurement2)∗100/measurement1|] of the same bone for each method were calculated.

Analysis of the data was done using MEDCALC 9.2 software (MedCalc Software, Mariakerke, Belgium). Passing and Bablok regression analysis was run to evaluate the agreement between the two methods [6, 7] and Bland and Altman plots were created for all comparisons. Precision and accuracy of the measurements were tested using the concordance correlation coefficient [8]. Paired samples *t*-test was used for the comparison of the absolute relative deviations between the two methods. The same test was also run to identify the significance of the differences between the bone lengths obtained by the two methods and to determine the significance of the differences between the lengths of the medial and lateral bones in metacarpal and metatarsals. A significance level of *P* ≤ 0.05 was used for all comparisons.

## 3. Results and Discussion

**Figure 2:**
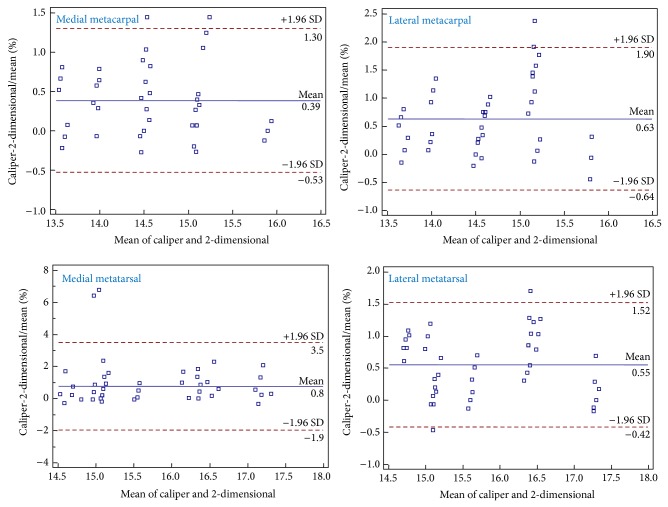
Bland-Altman plots showing the difference between bone lengths (cm) of sheep obtained with the caliper and the 2-dimensional method plotted against the mean of the 2 methods.

**Figure 3:**
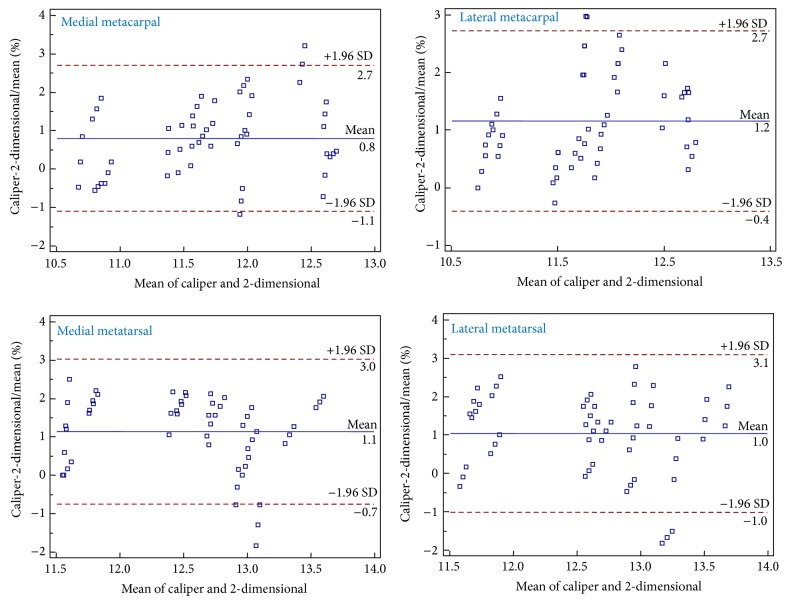
Bland-Altman plots showing the difference between bone lengths (cm) of goats obtained with the caliper and the 2-dimensional method plotted against the mean of the 2 methods.

The regression analysis according to Passing and Bablok revealed the equations presented in Table 1 between the metapodial lengths obtained with the caliper and the 2-dimensional method. The analysis of Bland and Altman plots for sheep (Figure 2) and goats (Figure 3) indicates that the two methods are linear and the 2-dimensional method is adequate for the measurement of the lengths of metacarpal and metatarsal bones in these animal species. The precision was over 97% for all bones in both sheep and goats (Table 1) confirming the strong linear relationship between the metapodial lengths measured by the two methods. The accuracy represents a bias correction factor, which measures the deviation of the best-fit line from the 45° line through the origin. In the present study the accuracy was over 97% in all comparisons indicating that the best-fit line almost fits the 45° line.

As it is shown in Table 2, the absolute relative deviations of the caliper measurements were significantly higher compared to those obtained by the 2-dimensional method in all bones (*P* < 0.05). This is indicative that the use of the 2-dimensional method for the measurements of these lengths is associated with lower error. This is probably due to the special anatomy of the proximal articular surfaces that does not allow a stable positioning of the caliper. The length of all bones measured with a caliper was significantly higher compared to those measured with the 2-dimensional method (*P* < 0.05; Table 3), suggesting that the manual method overestimates the bone lengths.

A complementary objective of the present study was to compare the lengths of medial and lateral metapodial bones in sheep and goats. Based on the aforementioned conclusion, the measurements obtained with the 2-dimensional method were used for these comparisons. As it is shown in Table 4, the lateral length of both metacarpals and metatarsals was significantly higher than medial length (*P* < 0.05) in goats, whereas in sheep the lateral length was significantly higher compared to medial one only in metatarsal bones (*P* < 0.05). According to our knowledge, there are few data in the available literature about the morphometrical characteristics of the metapodials which draw attention to the differences of lateral and medial bone length [2, 9] in artiodactyls. Páral et al. [9] measured the metapodial bones of both slaughtered and excavated bovines. The authors found that the lateral metatarsal was constantly longer than the medial metatarsal. Regarding the metacarpals, they reported that in some cases the medial metacarpal was longer and vice versa in others. In several cases the two bones were equal in length. Similar observations were recorded by Nacambo et al. [2]. The authors reported that the lateral sides of the metatarsal bones were longer than the medial sides. A smaller but significant difference was seen in the metacarpal bones. In the latter, in some bones the lateral side was longer whereas, in some others, the medial side was longer and in some others both sides were the same.

The asymmetry of the metapodial bones combined with the asymmetry of the digital bones [4, 10, 11] might affect the biomechanics of the foot in these species.

## 4. Conclusions

The main conclusion of the present study is that the 2-dimensional method is convenient and more accurate for the measurement of the metapodial bones' length in comparison with the caliper and that there is length asymmetry of the metapodials in the sheep and goats.

## Figures and Tables

**Table 1:** Passing and Bablok regression equations between the metapodial lengths obtained with the caliper (*y*) and the 2-dimensional method (*x*) with 95% confidence interval (CI) and precision and accuracy between the lengths obtained by the two methods in sheep and goats.

Metapodial bones	Passing and Bablok regression equations	Precision (%)	Accuracy (%)
Medial metacarpal sheep	*y* = 0.335 (CI: −0.862–0.868) + 0.980 (CI: 0.934–1.064)*x*	99.61	99.64
Lateral metacarpal sheep	*y* = −0.431 (CI: −2.441–0.791) + 1.035 (CI: 0.949–1.177)*x*	99.00	98.89
Medial metatarsal sheep	*y* = −0.212 (CI: −1.046–0.613) + 1.018 (CI: 0.966–1.071)*x*	99.82	99.63
Lateral metatarsal sheep	*y* = 0.075 (CI: −0.674–1.069) + 1.000 (CI: 0.938–1.048)*x*	99.66	99.49
Medial metacarpal goats	*y* = −1.211 (CI: −2.220–0.292) + 1.111 (CI: 0.982–1.200)*x*	98.60	98.68
Lateral metacarpal goats	*y* = −0.595 (CI: −1.897–0.336) + 1.062 (CI: 0.980–1.170)*x*	99.09	97.44
Medial metatarsal goats	*y* = 0.500 (CI: −0.297–1.670) + 0.976 (CI: 0.881–1.039)*x*	98.33	97.38
Lateral metatarsal goats	*y* = 0.165 (CI: −1.147–1.433) + 1.000 (CI: 0.897–1.102)*x*	97.95	97.89

**Table 2:** Mean ± SE of the absolute relative deviations (%) obtained between the two measurements of the same bone for each method (caliper and 2-dimensional method).

Metapodial bones	Caliper	Two-dimensional method
Medial metacarpal sheep	0.59 ± 0.03^a^	0.24 ± 0.02^b^
Lateral metacarpal sheep	0.60 ± 0.023^a^	0.24 ± 0.016^b^
Medial metatarsal sheep	0.61 ± 0.013^a^	0.25 ± 0.015^b^
Lateral metatarsal sheep	0.59 ± 0.014^a^	0.25 ± 0.016^b^
Medial metacarpal goats	0.72 ± 0.035^a^	0.31 ± 0.024^b^
Lateral metacarpal goats	0.72 ± 0.032^a^	0.29 ± 0.019^b^
Medial metatarsal goats	0.75 ± 0.020^a^	0.30 ± 0.013^b^
Lateral metatarsal goats	0.74 ± 0.018^a^	0.30 ± 0.017^b^

^a,b^Different superscripts at the same row denote significant difference.

**Table 3:** Mean ± SE of the lengths (cm) of metapodial bones in sheep and goats measured with the caliper and the 2-dimensional method.

Metapodial bones	Caliper	Two-dimensional method
Medial metacarpal sheep	14.61 ± 0.19^a^	14.55 ± 0.19^b^
Lateral metacarpal sheep	14.67 ± 0.18^a^	14.58 ± 0.18^b^
Medial metatarsal sheep	15.77 ± 0.24^a^	15.70 ± 0.24^b^
Lateral metatarsal sheep	15.86 ± 0.24^a^	15.77 ± 0.24^b^
Medial metacarpal goats	11.76 ± 0.15^a^	11.67 ± 0.15^b^
Lateral metacarpal goats	11.85 ± 0.15^a^	11.71 ± 0.15^b^
Medial metatarsal goats	12.62 ± 0.15^a^	12.47 ± 0.15^b^
Lateral metatarsal goats	12.70 ± 0.15^a^	12.57 ± 0.15^b^

^a,b^Different superscripts at the same row denote significant difference.

**Table 4:** Mean ± SE of the lengths (cm) of medial and lateral metapodial bones in sheep and goats measured with the 2-dimensional method.

Metapodial bones	Medial	Lateral
Metacarpal sheep	14.55 ± 0.19^a^	14.58 ± 0.18^a^
Metatarsal sheep	15.70 ± 0.24^a^	15.77 ± 0.24^b^
Metacarpal goats	11.67 ± 0.15^a^	11.71 ± 0.15^b^
Metatarsal goats	12.47 ± 0.15^a^	12.57 ± 0.15^b^

^a,b^Different superscripts at the same row denote significant difference.
